# Thermally Robust Solvent-Free Liquid Polyplexes for Heat-Shock Protection and Long-Term Room Temperature Storage of Therapeutic Nucleic Acids

**DOI:** 10.1021/acs.biomac.4c00117

**Published:** 2024-04-29

**Authors:** Yiyan Chen, Xiaoyan Lin, Xuhan Liu, Yifan Liu, Liem Bui-Le, Anna K. Blakney, Jonathan Yeow, Yunqing Zhu, Molly M. Stevens, Robin J. Shattock, Rongjun Chen, Alex P. S. Brogan, Jason P. Hallett

**Affiliations:** † Department of Chemical Engineering, Imperial College London, Exhibition Road, London SW7 2AZ, U.K.; ‡ Shenzhen University General Hospital, Shenzhen University Clinical Medical Academy, Shenzhen University, No. 1098 Xueyuan Avenue, Shenzhen 518000, P. R. China; § Department of Infectious Disease, Imperial College London, Norfolk Place, London W2 1NY, U.K.; ∥ School of Biomedical Engineering, Michael Smith Laboratories, 2185 East Mall, Vancouver, British Columbia V6T 1Z4, Canada; ⊥ Department of Materials, Department of Bioengineering, and Institute of Biomedical Engineering at Imperial College London, Prince Consort Rd, SW7 2AZ London, South Kensington, U.K.; # School of Materials Science and Engineering, Tongji University, Shanghai 200092, China; ∇ Department of Chemistry, King’s College London, 7 Trinity Street, London SE1 1DB, U.K.

## Abstract

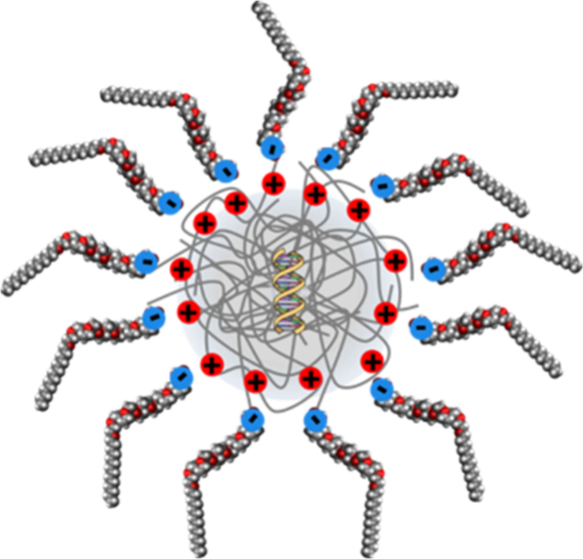

Nucleic acid therapeutics have attracted recent attention as promising preventative solutions for a broad range of diseases. Nonviral delivery vectors, such as cationic polymers, improve the cellular uptake of nucleic acids without suffering the drawbacks of viral delivery vectors. However, these delivery systems are faced with a major challenge for worldwide deployment, as their poor thermal stability elicits the need for cold chain transportation. Here, we demonstrate a biomaterial strategy to drastically improve the thermal stability of DNA polyplexes. Importantly, we demonstrate long-term room temperature storage with a transfection efficiency maintained for at least 9 months. Additionally, extreme heat shock studies show retained luciferase expression after heat treatment at 70 °C. We therefore provide a proof of concept for a platform biotechnology that could provide long-term room temperature storage for temperature-sensitive nucleic acid therapeutics, eliminating the need for the cold chain, which in turn would reduce the cost of distributing life-saving therapeutics worldwide.

## Introduction

Conventional vaccines, such as live-attenuated or deactivated virus vaccines and subunit vaccines, are a critical aspect of modern preventative medicine, saving millions of lives through the prevention of sustained outbreaks of infectious diseases and contributing to the eradication of many life-altering diseases.^1,2^ In recent years, nucleic acids have emerged as an attractive alternative to prevent and treat infectious diseases.^2−5^ Nucleic acid vaccines have several advantages over conventional vaccines as their versatility and relative simplicity allow for rapid development, potentially low cost and scalable production, as well as rapid deployment in response to emerging outbreaks.^1,2,4−6^ Additionally, nucleic acids exhibit great potential in gene therapy solutions for a range of noninfectious diseases including genetic disorders such as adenosine deaminase deficiency,^7^ cystic fibrosis,^8^ hemophilia,^9^ as well as various cancers.^10−14^ However, due to their susceptibility to degradation in the extracellular environment in combination with difficulties crossing cell membranes as a result of their relatively large sizes and overall negative charge, clinical translation of nucleic acid therapies remains a significant challenge.^15−17^ To both protect nucleic acids from enzymatic degradation and promote cellular uptake, nucleic acids typically require a delivery vehicle such as viral vectors or nonviral carriers.^18−22^ Viral vectors utilize the machinery of otherwise inactive viruses to deliver the therapeutic agent,^12,23^ whereas nonviral delivery vehicles are synthetic systems that aid in passage across cell membranes through a variety of mechanisms.^24^ The COVID-19 pandemic has brought nucleic acid delivery vectors to the mainstream via the now ubiquitous AstraZeneca^25^ viral vector-based vaccine as well as the nonviral-based Moderna^26^ and Pfizer-BioNTech^27,28^ mRNA vaccines. Nonviral carriers are generally regarded as safer delivery systems compared to viral vectors, as the latter have been known to induce unwanted immune responses and adverse effects.^18,29^ Moreover, nonviral delivery systems are usually easier to chemically modify and scale up production. This coupled with the potential to deliver large genetic payloads makes nonviral delivery vehicles favorable options over the viral alternatives.^18,30^

Typically, nonviral delivery systems are based on either cationic lipids or cationic polymers, which complex with nucleic acids through electrostatic interactions to form lipoplexes and polyplexes, respectively. Additionally, lipid nanoparticles (LNPs) have gained increasing attention for nonviral delivery.^31^ Despite their similarities, the mechanisms of cellular delivery are thought to be quite different. One of the main proposed mechanisms for lipoplex cargo delivery into the cytosol is through a process involving endosomal membrane displacement.^32^ Conversely, the suggested mechanism for polyplex delivery relies on the so-called “proton sponge” effect.^33^ In this case, the amine groups that make up the cationic polymers become protonated in the endosome, creating an osmotic force that leads to the rupture of the endosome and subsequent release of the polyplex into the cytosol.^34,35^ Lipoplexes have excellent biodegradability and low immunogenicity and are capable of delivering large DNA molecules. However, low colloidal stability of lipoplexes results in short half-lives^36^ and interactions between the lipids of the lipoplex and those of the cell membrane affect transfection efficiency.^37^ Polyplexes, on the other hand, have higher colloidal stability offering greater stability and more predictable interactions with membranes that allow for greater efficiencies.^37^ Many different polymers have been used for polyplex formation such as chitosan,^38^ polylysine,^39^ polyamino esters,^40^ and polyethylenimine (PEI).^41^ PEI is the most commonly used polymer carrier because it is stable to aggregation under physiological buffer conditions and possesses strong pH buffering capability over a wide pH range.^42^ Recently, poly(cystamine bis(acrylamide)-*co*-4-amino-1-butanol) (pABOL) has been shown to be a reducible and biodegradable alternative to PEI with greater transfection efficiency and lower cytotoxicity.^43^

The major challenge for the deployment of biomolecule-based therapeutics such as vaccines, particularly in resource-limited environments, is the requirement for the cold chain.^44,45^ Proteins and nucleic acids are prone to aggregation and degradation at room temperature and therefore need to be kept refrigerated or frozen at all times, from manufacturing to administration. The cold chain, which accounts for up to 80% of the cost of vaccination programs,^46^ poses a risk to the efficacy of the vaccines and severely limits the distribution of advanced therapeutics to regions with little or no refrigeration facilities.^44,45,47^ Despite providing protection in vivo, therapeutic delivery systems suffer from low stability and, therefore, limited shelf life. Particularly, nucleic acid polyplexes are prone to aggregation in aqueous solutions, resulting in reduced transfection efficiency.^48−50^ This requires nucleic acid polyplexes to either be lyophilized, stored under cryogenic temperatures, or prepared freshly prior to administration, all of which hinders clinical practicability.^49^

Several strategies have been introduced to overcome the poor stability of nucleic acid polyplexes, including lyophilization,^51^ the addition of sugars and other stabilizers to formulations,^49^ silica coatings,^52^ and novel design of polymers.^53,54^ However, most technologies do not improve the thermal stability sufficiently, and refrigerated storage at 2–8 °C is still required.^55^ It has been reported that the addition of sugars to lyophilized formulations can provide adequate room temperature storage conditions; however, the extraordinarily high sugar to DNA weight ratio (typically 1000–10,000) required for sufficient protection^49,56^ can present a significant risk to patients with complex dietary requirements such as diabetes.^57^ To realize the full potential of nucleic acid therapeutics, there is, therefore, significant demand for effective stabilization technologies for nucleic acid delivery vehicles that can allow for both facile manufacturing and room temperature storage.

Solvent-free proteins, a new class of biomaterial, have exhibited great potential in the stabilization of proteins and enzymes against temperature, aggregation, and nonaqueous environments through the formation of a polymer surfactant coronal layer on the surface of the biomolecules.^58^ Solvent-free liquids of proteins,^59^ enzymes,^60^ viruses,^61^ and antibodies^62^ have been successfully synthesized and shown to significantly increase the thermal stability by up to 100 °C. In all cases, the secondary and tertiary structure, as well as the biological function of the biomolecules, have been preserved in the absence of water.^63−65^ Given the thermal stability and retention of biological function observed for protein liquids, we were interested to see whether the same principle could be applied to stabilize single-particle nucleic acid delivery vehicles. Specifically, combining polyplexes with solvent-free liquids could provide a promising new strategy for constructing nucleic acid delivery vehicles with enhanced thermal tolerance for both long-term storage and heat-shock protection.

Here, we demonstrate for the first time the formation of solvent-free biofluids of DNA polyplexes resulting in a DNA-enriched biomaterial with high thermal robustness. Using a previously reported^43,66^ model polyplex system (DNA-P) comprised of a plasmid DNA encoding firefly luciferase condensed with a cationic polymer, pABOL, we show that DNA polyplex-surfactant biofluids (DNA–P-S) retain transfection efficacy for at least 9 months after storage at room temperature. Furthermore, we show that the biomaterial protects the nucleic acid against extreme heat shock, with the transfection efficiency of the polyplex biofluids remaining unchanged after exposure to 70 °C for 4 days. As such, the results shown here represent the first steps toward a biomaterial strategy for long-term room temperature storage of nucleic acid therapeutics. The result of this could drastically improve the deployment of advanced therapeutics worldwide, reducing the cost and response time for tackling large infectious disease outbreaks.

## Experimental Section

### DNA Oxidation of the Surfactant Brij S100

Brij-S100 (2.00 g) was dissolved in 50 mL of water and heated to 50 °C. Sodium bromide (516 mg), 2,2,6,6-tetramethyl-1-piperidinyloxy (TEMPO) (52 mg), and sodium hypochlorite (10 mL, available chlorine 6–14%) were added to the stirring hot aqueous Brij-S100 solution. The pH of the solution was increased to above pH 11 with aqueous sodium hydroxide (1 M), which made the solution turn from yellow to colorless, and the solution was subsequently stirred for 24 h. The reaction had turned green after 24 h and was then quenched with ethanol, resulting in a colorless solution. The pH was reduced to below pH 1 with aqueous hydrochloric acid (1 M), causing the solution to turn yellow. The product was extracted with chloroform (3 × 80 mL) and combined. The chloroform layers were dried on a rotary evaporator and then dissolved in hot ethanol (50 mL). The ethanol solution was left in the freezer at −20 °C overnight for the product to crystallize. The supernatant was removed with a syringe, and then, the crystallized product was dissolved in ethanol and dried on the rotary evaporator. The waxy solid was dissolved in water and then lyophilized for 48 h resulting in a brown waxy solid (1.83 g, 91.3%). FTIR and NMR spectroscopy confirmed the oxidation. FTIR in Figure S1A showed the presence of the C=O stretching vibration band of carboxylic acids at 1755 cm^–1^.^67^ Also, ^13^C NMR showed the presence of a carboxylic C=O at 171 ppm, which was not present in Brij-s100 solution before the oxidation (Figure S1b).

### Preparation of the DNA–P-S Biofluids

Stock solution of 100 kDa pABOL(Chemical structure provided in Figure S2) was prepared by dissolving the polymer in ultrapure water at 25 mg/mL and filtered using 0.2 μm syringe filters. Stock solution of the surfactant was obtained by diluting the oxidized Brij S100 in water at 10 mg/mL followed by pH adjustment to 7.0. DNA polyplexes were prepared using the established titration method with a polymer to DNA mass ratio of 45:1 (N/P ratio = 38.94).^43^ 100 μL of 6732 bp (MW 4443 kDa) gWiz-Luc DNA (50 μg/mL) and 400 μL of pABOL (562.5 μg/mL) were added to separate centrifuge tubes. The tube containing pABOL was placed on a stir plate and stirred at 1200 rpm. The DNA was added to the pABOL by using an Aladdin Single-Syringe Pump (World Precision Instruments) at a pumping rate of 160 μL/min to form the DNA-pABOL polyplexes (DNA-P). The freshly prepared DNA-P was slowly added to the anionic surfactant at a stirring rate of 250 rpm and a molar charge ratio of 2:1. The sample was stirred overnight, followed by purification with 0.45 μm syringe filters and then concentrated using a Thermo Scientific Piers Protein Concentrator PES (MWCO 10k) at 40,000 g for 30 min. The DNA–P-S conjugates were lyophilized for 48 h, annealed at 60 °C for 30 min, and then finally cooled to room temperature yielding the DNA–P-S biofluids.^65^ For the room temperature storage studies, the DNA-P was stored at room temperature for 8 weeks, and the DNA–P-S biofluids were stored at room temperature for 9 months. For the heat shock studies, DNA-P and DNA–P-S biofluid were heated on a hot plate at 70 °C for 4 days, and a comparison group of DNA-P and DNA–P-S from the same batch were stored in the fridge at 4 °C for 4 days. The DNA–P-S biofluids were resuspended in water prior to characterization and reconstituted in PBS buffer for the in vitro transfection studies.

### In Vitro Transfection Studies

HEK 293 (human embryonic kidney cell line) cells were obtained from the American Type Culture Collection and cultured in high glucose DMEM medium supplemented with 10% fetal bovine serum (FBS) and 1% (v/v) penicillin/streptomycin in a humidified atmosphere with 5% CO_2_ at 37 °C. For the transfection assay, HEK 293 cells were seeded in 96-well plates at a density of 5 × 10^4^ cells per well 24 h before transfection to reach 60–80% confluence. Following the removal of the growth medium, 100 μL of fresh serum-free DMEM medium was added per well, and 10–20 μL of DNA samples were subsequently added in replicates of 5. After 4 h of incubation, the transfection medium was replaced with the growth medium, which was supplemented with 10% FBS and 1% (v/v) penicillin/streptomycin. After 24 h from the initial transfection, 50 μL of medium from transfected cells was taken from each well and assayed with 50 μL of ONE-Glo luciferase substrate. The luciferase activity was analyzed using a Labtech FLUOstar microplate reader and expressed as relative light units.

## Results and Discussion

### Characterization of the DNA-P and DNA–P-S

**Figure 1:**
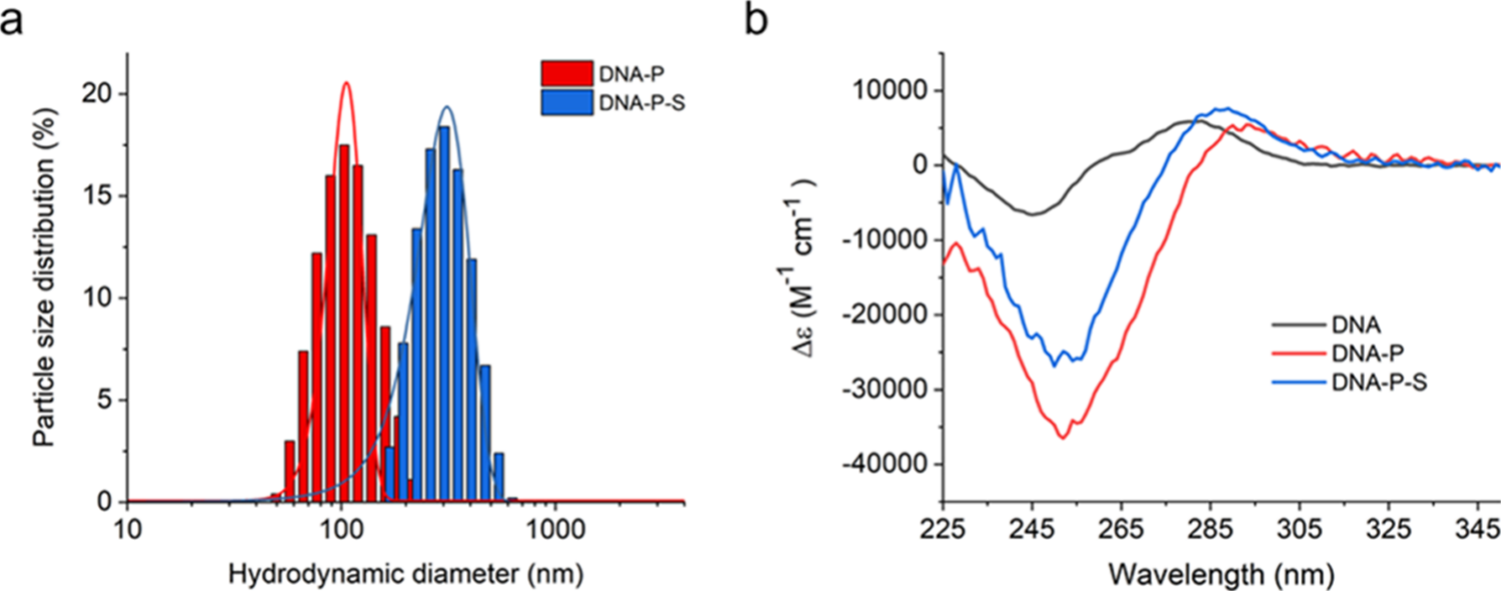
(a) Plot of particle size distribution by intensity as measured by DLS against hydrodynamic diameter for DNA-P (red) and DNA–P-S (blue), size distributions fitted with Gaussian distributions to calculate mean hydrodynamic diameters. (b) Circular dichroism (CD) measuring molar extinction of DNA (black), DNA-P (red), and DNA–P-S (blue) at room temperature.

Following similar procedures to solvent-free liquid proteins,^58^ DNA–P-S was assembled via a 2-step process. Successful assembly of the DNA–P-S conjugates was confirmed using dynamic light scattering (DLS), which showed an increase in the hydrodynamic diameter of the DNA-P from 113 ± 34 to 303 ± 85 nm (Figure 1a). Additionally, the zeta potential of the DNA-P decreased from 62.3 ± 2.6 to −9.7 ± 2.9 mV upon formation of DNA–P-S, confirming stoichiometric complexation of the negatively charged surfactant to DNA-P. The DNA polyplex-surfactant conjugates (DNA–P-S) were then freeze-dried and annealed at 60 °C to yield solvent-free DNA polyplex-surfactant biofluids (DNA–P-S biofluids). Attenuated total reflectance Fourier transform infrared spectroscopy (ATR-FTIR) confirmed the formation of the DNA–P-S by the presence of both pABOL and surfactant (Figure S3). Unfortunately, due to the low mass ratio of DNA to polymer, and overlapping pABOL peaks, FTIR was unsuitable for the characterization of the DNA structure.

We therefore turned to circular dichroism (CD) to assess the structure of DNA during DNA-P and DNA–P-S formation. The CD of the gWiz-Luc DNA exhibited the characteristic spectra of the B conformation of DNA with a positive peak at 280 nm and a negative peak at 245 nm (Figure 1b). The formation of DNA-P caused a shift in DNA structure to the A form, as evidenced by a red shift of the positive feature from 280 to 293 nm, in conjunction with a red shift in the negative feature from 245 to 253 nm that was concomitant with a 6-fold increase in the molar extinction from −6605 to −36589 M^–1^·cm^–1^ (Figure 1b). The shift in DNA structure was in agreement with previously reported structural changes that occur with the formation of DNA-PEI polyplexes.^68^ Upon complexation with the surfactant, the spectra of DNA–P-S showed no obvious changes in DNA structure (Figure 1b). This indicated that other than the expected shift from B to A structure upon polyplex formation, there were no further detectable structural changes in the DNA after complexation with the surfactant to form DNA–P-S.

### Thermal Stability of the DNA-P and DNA–P-S

**Figure 2:**
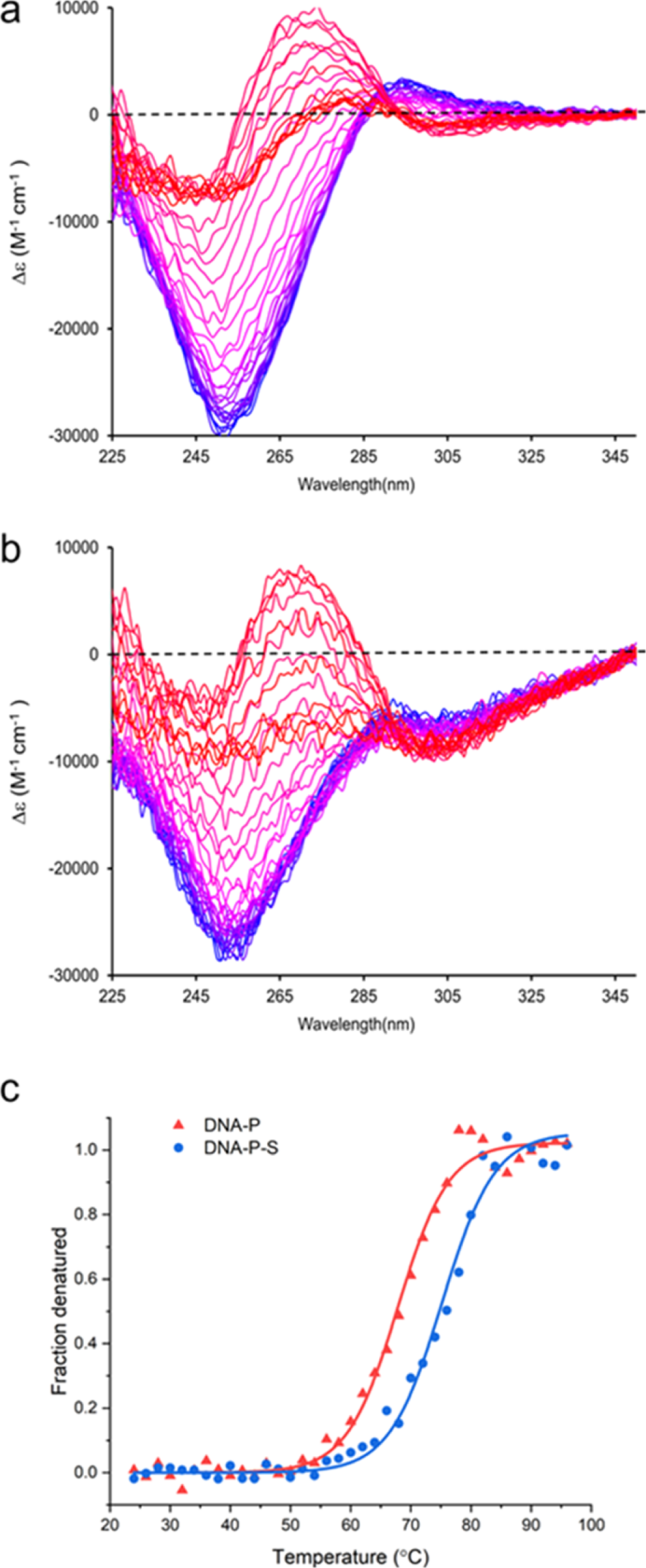
Temperature-dependent circular dichroism spectra showing thermal denaturation of (a) DNA-P and (b) DNA–P-S conjugates from 24 °C (blue) to 96 °C (red) at 2 °C intervals. (c) Plot of equilibrium fraction denatured as a function of temperature for the DNA-P and DNA–P-S as calculated from the data shown in Figure 2a,b.

Given that the formation of DNA–P-S did not significantly alter the structure of the DNA, we sought to establish whether stability had been altered. For this, we chose to investigate the thermal stability of the DNA in the polyplex and surfactant conjugate by using both temperature-dependent circular dichroism and UV–vis spectroscopy measurements. UV–vis showed that the denaturation of DNA started at 51 °C, with a half-denaturation temperature of 60 °C and a 42.6% increase in the UV–vis absorbance upon denaturation (Figure S4b,d). In comparison, the DNA-P demonstrated higher thermal stability according to UV–vis as the double helix structure persisted until 81 °C, which was followed by rapid denaturation at temperatures higher than 81 °C (Figure S4c,d). However, as we had already determined that the structure of DNA had changed upon polyplex formation, we used temperature-dependent CD to investigate further. Although UV–vis indicated that the DNA-P maintained the double helix structure at temperatures lower than 81 °C, CD revealed that the tertiary structure of the DNA-P gradually transformed from the B-form to the A-form (Figure 2a). This was indicated by the blue shift of the positive peak in CD spectra from 293 to 273 nm with a progressive increase in the peak intensity from 17,819° M^–1^·cm^–1^ at 24 °C to 52,002° M^–1^·cm^–1^ at 81 °C, resulting in a dominant positive band at 250–300 nm (Figure 2a).^69,70^ As the temperature further increased from 81 to 96 °C, the positive band shifted from 273 to 280 nm with a reduction in the peak amplitude to 23,805° M^–1^·cm^–1^. The shift of the crossover in the long wavelength position from 255 to 265 nm was consistent with the unwinding of the helix structure and the denaturation of the double-stranded DNA into single-stranded DNA.^69^ As such, this showed that the denaturation of DNA in the polyplex shifted to a two-stage transformation. Consistent with the observations of minimal changes in structure, the denaturation of DNA–P-S followed that of DNA-P, with similar spectra shifts as compared to DNA-P, showing conversion from B-form to A-form followed by denaturation upon heat treatment (Figure 2b). In order to assess the equilibrium denaturation thermodynamics of the DNA–P-S, the molar ellipticity of the characteristic negative CD peak at 250 nm was used as a parameter to determine the thermal stability of DNA in DNA-P and DNA–P-S.^59,71^ The resultant fraction denatured plots, representing the structural changes in DNA with temperature, showed a sigmoidal response of DNA structure to temperature (Figure 2c). From these plots, we were able to determine the thermal stability, via the half-denaturation temperatures, of DNA-P and DNA–P-S conjugates as 67 and 75 °C, respectively (Figure 2c). The 8 °C increase in thermal stability indicated that, while the structure of the DNA was largely unchanged upon formation of DNA–P-S, the surfactant coronal layer on the surface of the polyplex provided additional protection for the DNA from thermal denaturation. This was likely the result of an increased macromolecule crowding and confinement.^59,65^

### Transfection Studies and Room Temperature Storage of the DNA–P-S Biofluids

**Figure 3:**
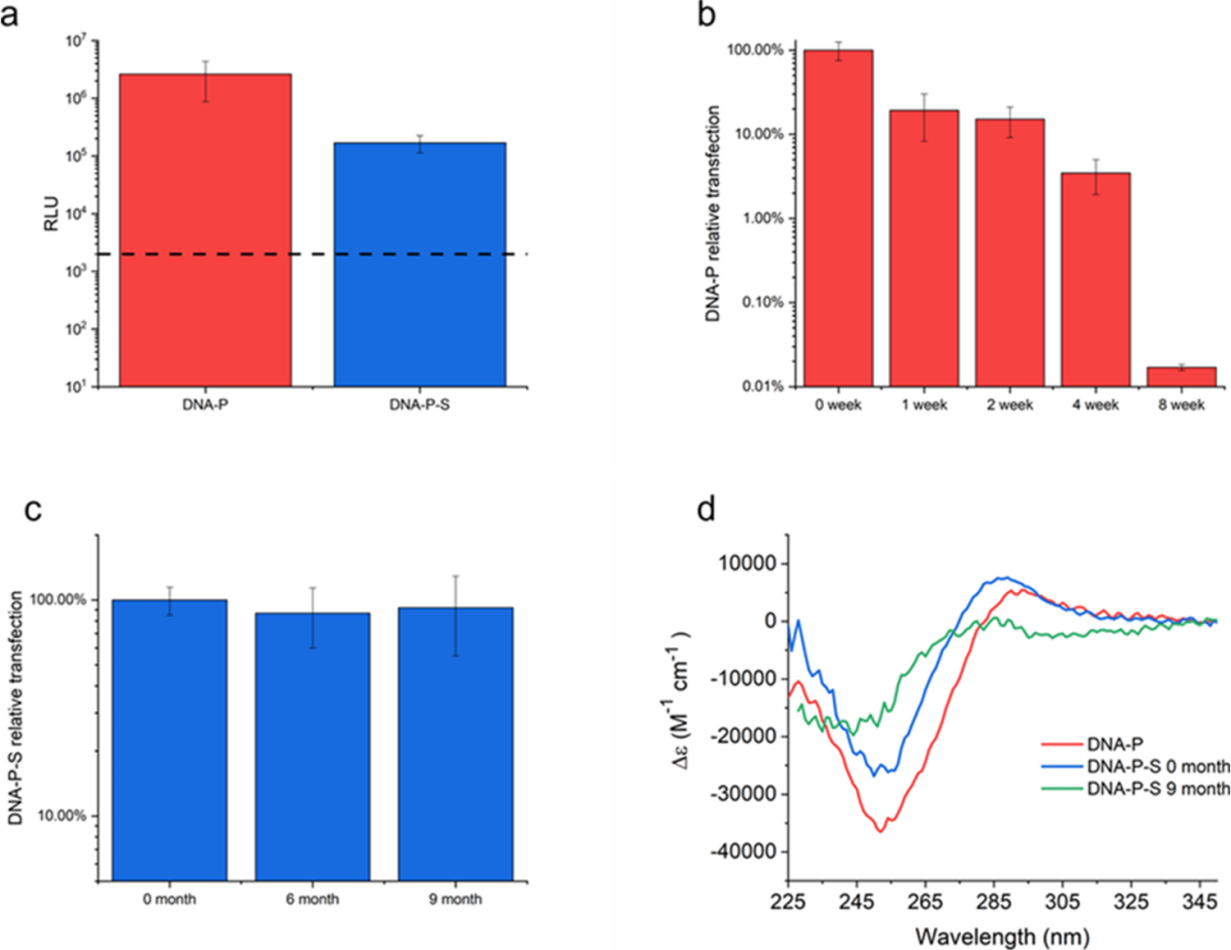
(a) Transfection efficiency (relative light units indicating expression of luciferase) of the freshly prepared DNA-P(red) and the DNA–P-S biofluids (blue); Data are shown as mean ± S.D., *n* = 5; the dose for transfection studies was 500 ng per well. The black dashed line represents the limit of detection for the luciferin-luciferase assay. (b) Relative transfection efficiency of the DNA-P with respect to 0 week transfection RLU after storage at room temperature. (c) Relative transfection efficiency for DNA–P-S biofluids (as compared to reference DNA-P made on the day of measurement to reduce the influence from cell conditions) with respect to 0 month after storage at room temperature. (d) CD spectra of DNA-P (red), DNA–P-S (blue) and redissolved DNA–P-S after 9-month storage at room temperature (green).

Having established that the DNA structure was maintained with some enhanced stability as a result of conjugate formation, we sought to determine whether the biological function of the nucleic acid remained. The model DNA used in this study, gWiz-Luc, encodes firefly luciferase such that successful transfection in cells will yield easily identifiable and characteristic luminescence when treated with an appropriate luciferin substrate. Transfection assays, performed with HEK 293 cells, showed that DNA–P-S retained high transfection ability, although there was a small reduction in the transfection efficacy when compared to DNA-P (Figure 3a). This likely reflected the slight decrease in the DNA structure observed for DNA–P-S in the CD spectra as well as a potential reduction in cell permeability resulting from the change in the surface charge of the DNA–P-S complex compared to the polyplex.

Long-term room temperature storage and, therefore, cold chain circumvention are long sought-after goals for vaccine development. It has been previously established that solvent-free liquids of proteins significantly enhance protein stability, resulting in greatly improved long-term stability.^62^ We, therefore, undertook a room temperature storage study comparing the transfection efficiency (as a marker for retained biological activity) of aqueous DNA-P with that of the DNA–P-S biofluid (Figure 3). DNA-P were stored at room temperature for 8 weeks, and the DNA–P-S biofluids were stored at room temperature for 9 months. The transfection efficiency of DNA-P gradually decreased over 4 weeks and after 8 weeks had fallen below the reliable detection limit of the luciferin-luciferase assay, indicating that the DNA had lost all meaningful transfection ability (Figure 3b). In comparison, DNA–P-S biofluids stored at room temperature showed little reduction in transfection ability after 9 months with >90% of relative transfection remaining (Figure 3c). Cell activity varied throughout the extended period (Figure S6), such that it became necessary to normalize absolute transfection efficiency to a control of freshly prepared DNA-P at each time point. Nevertheless, the results indicated that while aqueous DNA-P was not suitable for long-term storage at room temperature, the DNA–P-S biofluid showed remarkable long-term stability for storage up to at least 9 months. CD spectroscopy (Figure 3d) showed that after 9 months, DNA–P-S still had characteristic features at 245 and 280 nm, indicating retention of B-form structure. However, both features had reduced in intensity, suggesting that some change in the DNA tertiary structure was occurring. Temperature-dependent CD (Figure S7) revealed that this loss in structure was associated with a slight reduction in thermal stability, with thermal stability reducing slightly from 75 to 69 °C after 9 months at room temperature (Figure S8). Regardless, these results established that the DNA–P-S biofluids provided significant enhancement of the thermal stability and room temperature storage capability for nucleic acid polyplexes.

**Figure 4:**
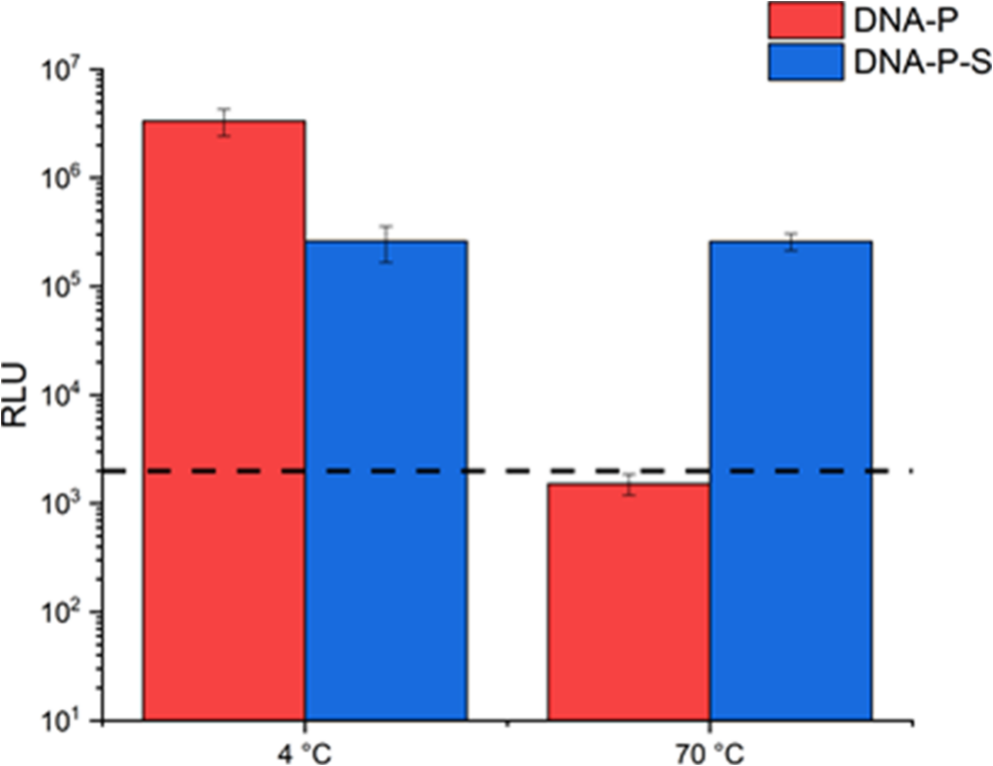
Transfection efficiency (relative light units) of DNA-P (red) and DNA–P-S biofluid (blue) after being stored at 4 °C for 4 days and after being heated at 70 °C for 4 days. Data shown as mean ± S.D., *n* = 5; the dose for transfection studies was 500 ng per well. The black dash represents the limit of detection for the luciferin-luciferase assay.

One of the major risks ensuring the viability of vaccines during transportation is protection not just against temperature but also fluctuations in temperature. Solvent-free liquid proteins have commonly exhibited hyperthermostability (frequently stable up to temperatures above 150 °C) and as such, we wanted to investigate whether this was also a feature of the DNA–P-S biofluids developed here. Consequently, we performed an exaggerated heat shock experiment to assess whether, as a storage medium, DNA–P-S biofluids would resist fluctuations in the temperature. For this, we compared the transfection of aqueous DNA-P and DNA–P-S biofluids after 4 days of incubation at 4 and 70 °C, respectively (Figure 4). After 4 days at 4 °C, both DNA-P and DNA–P-S retained the same level of transfection efficiency as freshly made samples (Figure 4). Upon heat treatment, the luciferase expression level of the DNA-P decreased by 3 orders of magnitude, indicating the denaturation of the DNA-P and the loss of transfection ability after incubation at elevated temperatures. Remarkably, the DNA–P-S biofluids retained the same level of transfection ability, with luciferase expression remaining unchanged. This result indicated that not only does DNA–P-S show potential for long-term cold chain-free storage but it can also protect the nucleic acid polyplex against elevated temperatures.

## Conclusions

In conclusion, we have demonstrated for the first time a biomaterial strategy for the combined stabilization and delivery of nucleic acids. DNA–P-S biofluids displayed high thermal tolerance, showing excellent potential to circumvent cold chain transportation issues by facilitating room temperature storage of therapeutic nucleic acids. Circular dichroism verified the retention of tertiary structure within DNA–P-S conjugates, and aqueous stability increased by 8 °C from 67 to 75 °C after the addition of the anionic surfactant. Although the transfection efficiency was slightly reduced by the addition of the anionic surfactant, the resultant biofluid preserved this biological function for 9 months after storage at room temperature. Additionally, thermal robustness was further demonstrated through an exaggerated heat shock trial, with luciferase expression remaining unchanged despite being exposed to 70 °C for 4 days. Equivalent experiments with just the DNA-P polyplexes showed complete loss of bioactivity; demonstrating the importance of the surfactant coronal layer of DNA–P-S in protecting the nucleic acid polyplexes. Long-term thermal stability of the DNA-P was thus significantly increased in the solvent-free biofluids, offering promising biotechnology to eliminate the cold chain for the transportation and storage of temperature-sensitive therapeutics. Future work will now move to develop this methodology for RNA-based therapeutics, thus providing an avenue for room temperature vaccine storage. Expansion to RNA-based technologies will also provide novel storage strategies for the nucleic acid therapeutics currently used in gene therapy. Ultimately, this biotechnology platform could enable long-term room temperature storage of therapeutic nucleic acids, eliminating the requirement for the cold chain. Such an outcome would drastically improve the accessibility of life-saving therapeutics in resource-limited areas and enable a more rapid, comprehensive, and equitable response to large disease outbreaks.

## Supporting Information Available

The Supporting Information is available free of charge at The following files are available free of charge. The Supporting Information is available free of charge at https://pubs.acs.org/doi/10.1021/acs.biomac.4c00117.

FTIR and NMR spectra of Brij-S100; supporting CD spectra and transfection results (PDF)
