# Supplementary material for: Thermally Robust Solvent-Free Liquid Polyplexes for Heat-Shock Protection and Long-Term Room Temperature Storage of Therapeutic Nucleic Acids
Source: Biomacromolecules. 2024 Apr 29;25(5):2965–72. doi: 10.1021/acs.biomac.4c00117 (PMC11094731; doi:10.1021/acs.biomac.4c00117)
Supplement: Supplementary file 1 — bm4c00117_si_001.pdf [file bm4c00117_si_001.pdf]

Supplementary Information

Thermally Robust Solvent-free Liquid Polyplexes for Heat-shock Protection and Long-Term Room Temperature Storage of Therapeutic Nucleic Acids

Yiyan Chen[1,†], Xiaoyan Lin[1,†], Xuhan Liu[1,2], Yifan Liu[1], Liem Bui-Le[1], Anna K. Blakney[3,4], Jonathan Yeow[5], Yunqing Zhu[6], Molly M. Stevens[5], Robin J. Shattock[3], Rongjun Chen[1], Alex P. S. Brogan[7,*], Jason P. Hallett[1,*]

1. Department of Chemical Engineering, Imperial College London, Exhibition Road, London, SW7 2AZ, UK.

2. Shenzhen University General Hospital, Shenzhen University Clinical Medical Academy, Shenzhen University, No. 1098 Xueyuan Avenue, Shenzhen, 518000, PR China

3. Department of Infectious Disease, Imperial College London, Norfolk Place, London, W2 1NY, UK

4. Michael Smith Laboratories, School of Biomedical Engineering, 2185 East Mall, Vancouver, BC, V6T 1Z4, Canada.

5. Department of Materials, Department of Bioengineering, and Institute of Biomedical Engineering at Imperial College London, Prince Consort Rd, SW7 2AZ London, South Kensington, UK

6. School of Materials Science and Engineering, Tongji University, Shanghai, 200092, China.

7. Department of Chemistry, King's College London, 7 Trinity Street, London, SE1 1DB, UK.

‡Y.C. and X.L. contributed equally to this work.

Materials

Chemicals were purchased from Sigma Aldrich and VWR and used without purification. The plasmid DNA gWiz-Luc was obtained from Aldevron, US; 100 kDa pABOL was synthesized using an established method.[1] DMEM medium, fetal bovine serum, penicillin and streptomycin were ordered from Gibco, UK. ONE-Glo Luciferase Assay System was obtained from Promega, UK.

Analytical Characterisation

Dynamic Light Scattering (DLS) characterisation was performed using a Malvern Zetasizer μV. Zeta potential measurements were conducted on a Malvern Zetasizer Nano. DNA samples were measured in disposable cuvettes at a concentration of 0.5 μg/mL in water. All

measurements were repeated three times.

FTIR spectra were obtained using an Agilent Cary 630 FTIR spectrometer from 400 to 4000 wavenumbers at 2 cm^{-1} resolution and with 32 scans per spectrum. Data was obtained and analyzed using Agilent MicroLab software.

UV-vis spectroscopy was performed on a Thermofisher Nanodrop One Spectrophotometer to determine the concentration of the DNA samples. Temperature-dependent UV-vis spectra were acquired on a Shimadzu UV2600 equipped with a Peltier-controlled heating module. Aqueous solutions of the DNA samples were measured in 10 mm quartz cuvettes from 24 °C – 96 °C at 3 °C intervals.

Circular Dichroism (CD) spectra (Figure 2) were acquired on a Jasco J-815 Spectropolarimeter fitted with a CDF-426S Peltier unit in Francis Crick Institution. CD spectra (Figure 3D and Figure S6) were acquired on an Applied Photophysics Chirascan Spectropolarimeter fitted with a Quantum Northwest Peltier temperature controller. The DNA samples were diluted with water to a concentration of 30 – 40 µg/mL and placed in a 10 mm quartz cuvette. Thermal denaturation curves of the DNA samples were obtained by recording the spectra every 3 °C in the temperature range of 24 °C – 96 °C. Spectra were acquired between 225 nm and 345 nm with an integration time of 2 s, a bandwidth of 2 nm and a step of 1 nm.

Figures

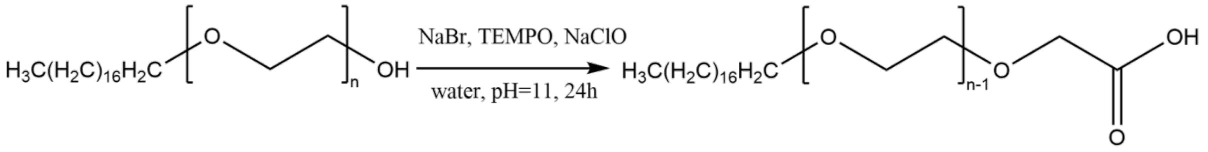

Scheme S1 Reaction scheme for the oxidastion of Brij S100 from alcohol to carbocylic acid. n~100.

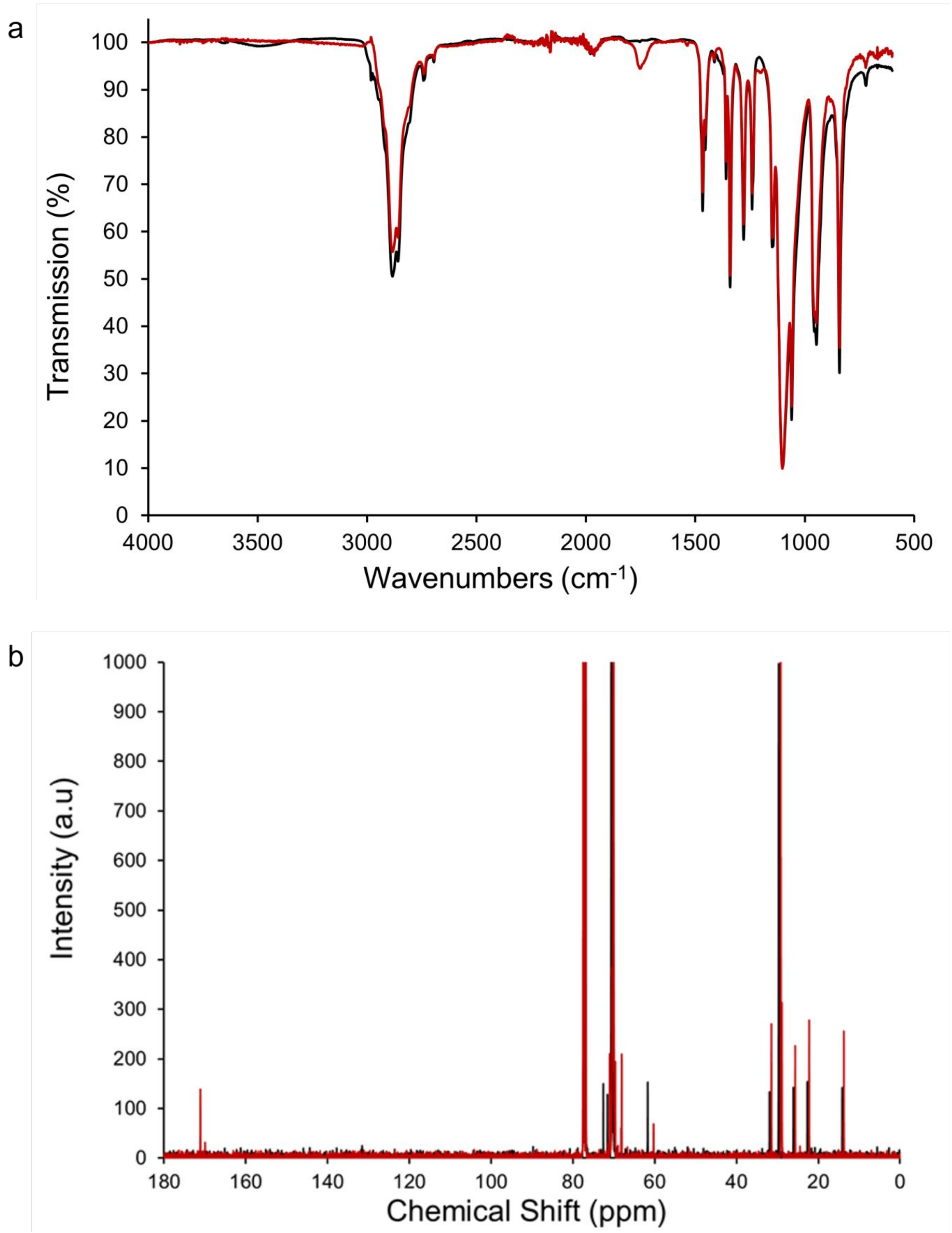

Figure S1 Comparison FTIR (**a**) and ^{13}C NMR (**b**) data between Brij-S100 surfactant before (black) and after oxidised (red).

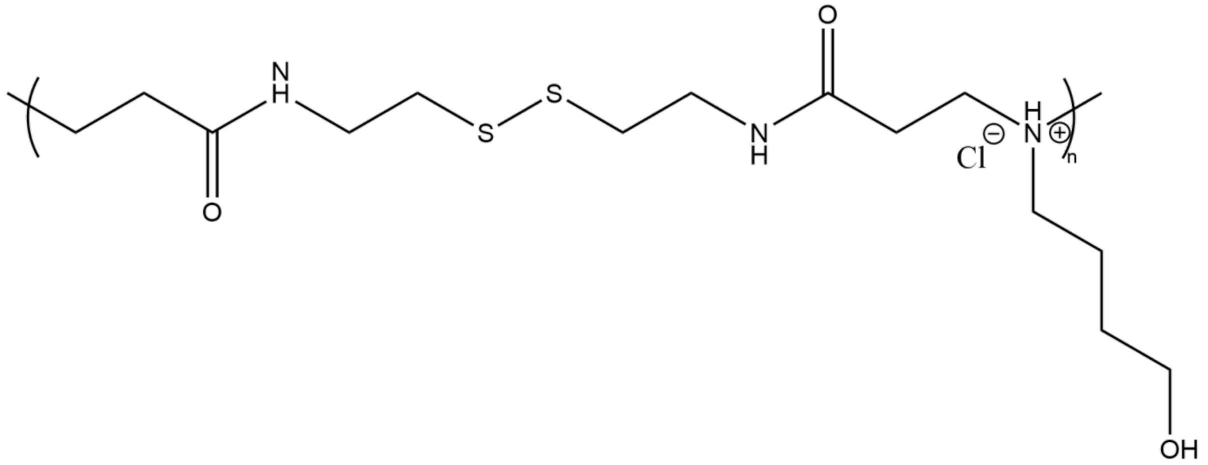

Figure S2 Structure of poly(CBA-co-4-amino-1-butanol (ABOL)) (pABOL). pABOL was prepared at molecular weight 100 kDa using optimized aza-Michael polyaddition synthesis protocol[1].

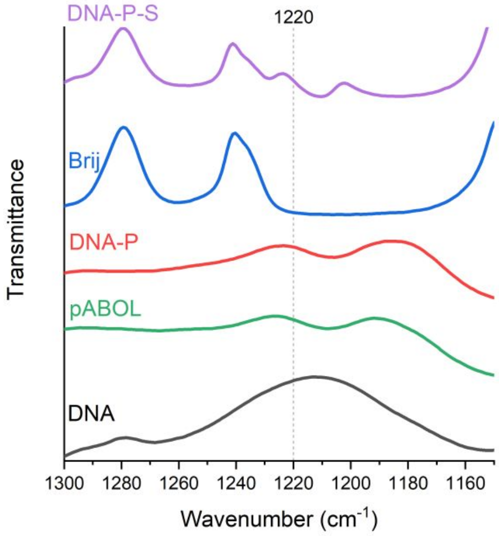

Figure S3 Comparison FTIR of DNA, pABOL, DNA-P, oxidised Brij S100 and DNA-P-S from 1150 to 1300 cm^{-1}.

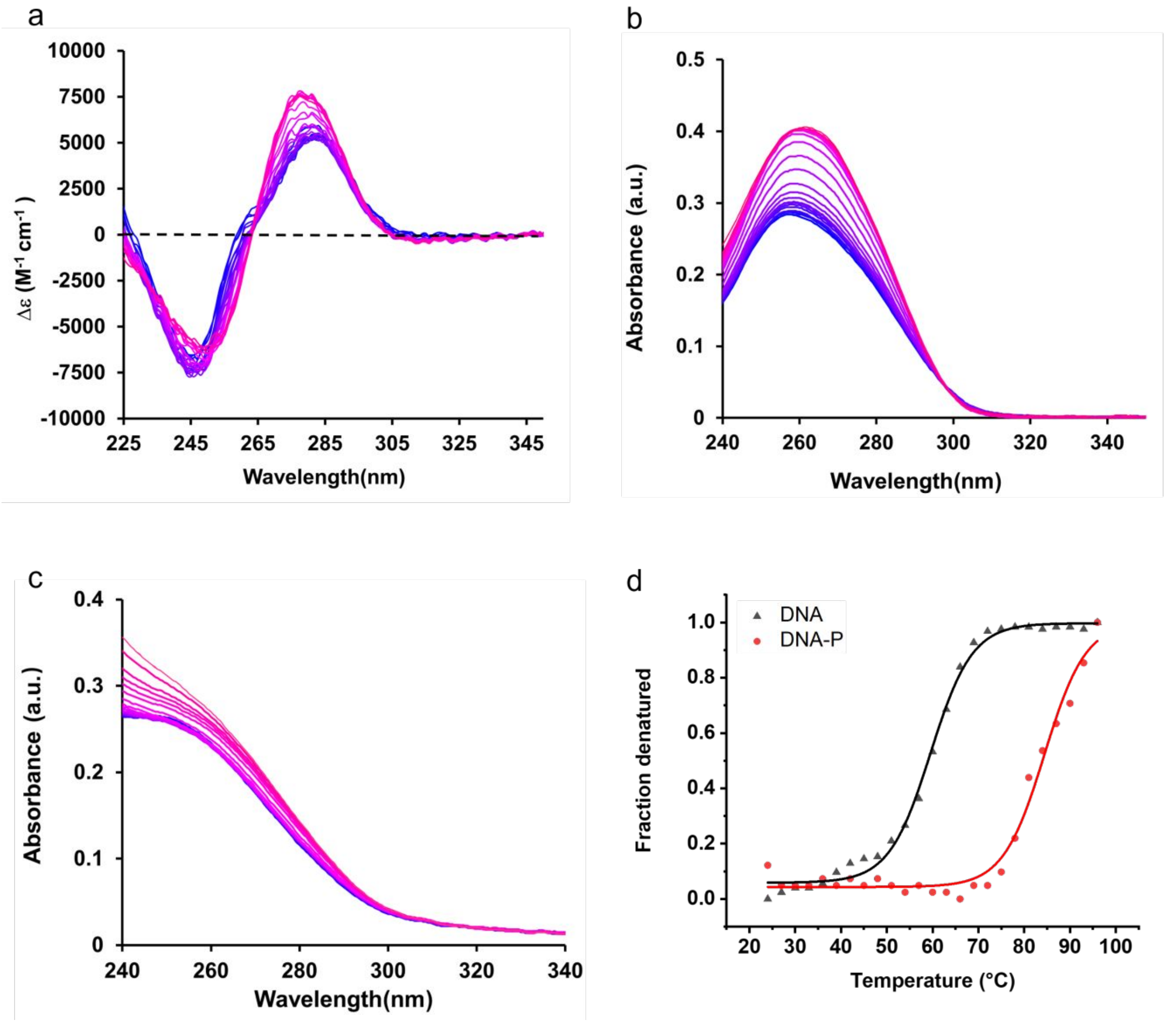

Figure S4 Temperature-dependent (**a**) Circular Dichroism and (**b**) UV-vis spectra of the DNA as the temperature increased from 24 °C to 96 °C at an interval of 3 °C; (**c**) Temperature-dependent UV-vis spectra of the DNA-P in the temperature range 24 °C – 96 °C at an interval of 3 °C; (**d**) Fraction denatured as a function of temperature for DNA and DNA-P based on the UV-vis absorbance at 260 nm.

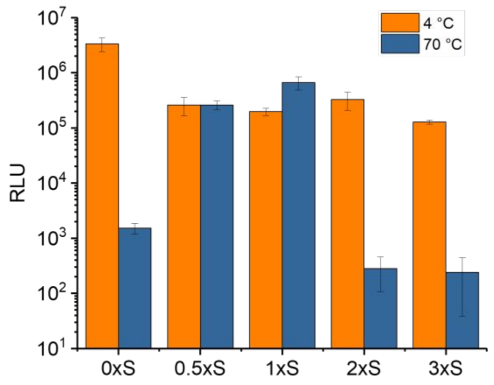

Figure S5 Transfection efficiency of DNA-P-S biofluids conjugated with different concentrations of surfactants after storage at 4°C and 70 °C for 4 days. Data shown as mean ± S.D., n=5; The dose for transfection studies was 500 ng per well; 1x is the molar ratio by charge of the oxidised Brij S100 added to the DNA polyplexes.

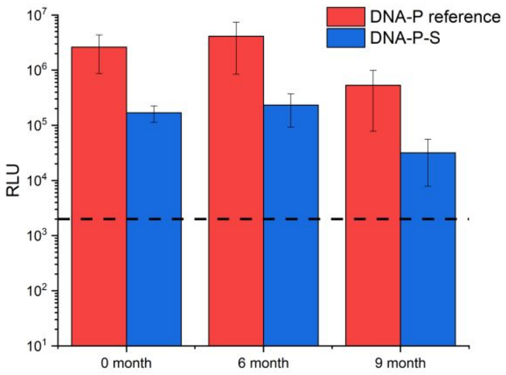

Figure S6 Transfection efficiency of freshly made DNA-P and DNA-P-S biofluids stored at room temperature at 0 months, 6 months and 9 months. Data shown as mean ± S.D., n=5; The dose for transfection studies was 500 ng per well.

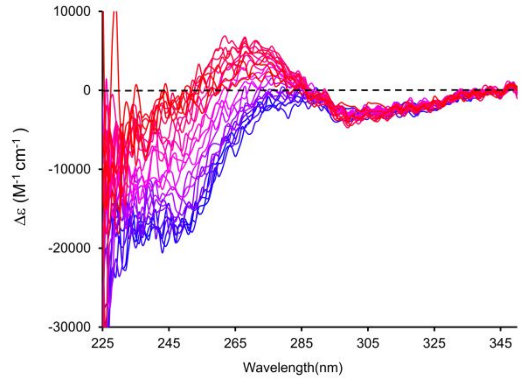

Figure S7 Temperature-dependent circular dichroism spectra of DNA-P-S biofluid after 9 months at room temperature.

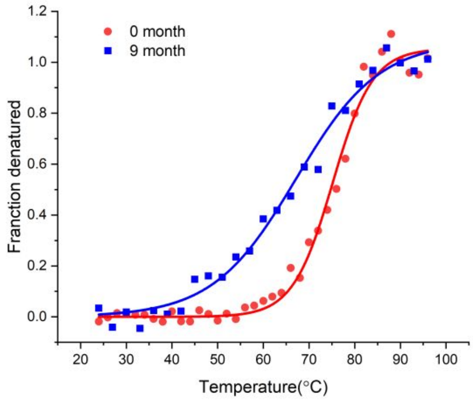

Figure S8 Plot of equilibrium fraction denatured as a function of temperature for DNA-P-S biofluid stored at room temperature at 0 months and 9 months as calculated from the molar ellipticity at 250 nm.

References

(1) Blakney, A. K.; Zhu, Y.; McKay, P. F.; Bouton, C. R.; Yeow, J.; Tang, J.; Hu, K.; Samnuan, K.; Grigsby, C. L.; Shattock, R. J.; et al. Big Is Beautiful: Enhanced saRNA Delivery and Immunogenicity by a Higher Molecular Weight, Bioreducible, Cationic Polymer. *ACS Nano* **2020**, *14* (5), 5711-5727. DOI: 10.1021/acsnano.0c00326.
